# S221. QUANTITATIVE SYSTEMS PHARMACOLOGY AS AN ALTERNATIVE TO CHLORPROMAZINE EQUIVALENTS: PREDICTIVE VALIDATION FROM A CRIS DATABASE EXPERIMENT

**DOI:** 10.1093/schbul/sby018.1008

**Published:** 2018-04-01

**Authors:** Hugo Geerts, Athan Spiros, Giouliana Kadra, Robert Stewart, Richard Hayes, Hitesh Shetty, Ehtesham Iqbal

**Affiliations:** 1 In Silico Biosciences; 2 Institute of Psychiatry, King’s College London; 3 BRC Nucleus, South London and Maudsley NHS Foundation Trust; 4 BRC Nucleus, South London and Maudsley

## Abstract

**Background:**

Polypharmacy is common in real clinical practice and in pharma-sponsored clinical trials. Chlorpromazine equivalents do not take into account pharmacodynamic interactions of drug combinations. If there is a sufficiently deep calibration set available, bio-informatics approaches can build classifiers for clinical phenotypes. However, this is not always the case which severely limits the generalizability of the predictions.

**Methods:**

We applied a mechanism-based computer model of a cortico-striatal-thalamocortical loop of the dorsal motor circuit that has been calibrated with clinical data on the prevalence of extrapyramidal symptoms after antipsychotic treatment in schizophrenia patients and therapeutic interventions in Parkinson’s patients[1]. The Quantitative Systems Pharmacology (QSP) model is based on the appropriate connections between basal ganglia regions and consists of 220 neurons (8 different cell types), 3500 synapses and implementations of 32 CNS active targets, based on their unique locations and coupling with intracellular pathways. Modulation of the various CNS targets were calculated on simulating the competition between the endogenous neurotransmitter and the two drugs at their appropriate concentrations and affinity.

The model was challenged to blindly predict the extrapyramidal symptoms liability of 1,124 patients prescribed two antipsychotics for six or more months (772 unique combinations). Anonymized data were derived from South London and Maudsley NHS Foundation Trust (SLAM) electronic health records (EHR). Extrapyramidal side effects were captured and identified using a combination of Natural Language Processing and a bespoke algorithm [2]. Only names and doses of the two drugs were made available without any calibration set.

**Results:**

Blind prediction of the outcomes using a Receiver Operating Characteristic curve with the QSP model resulted in an Area-Under-the Curve of 0.64 (p<0.01), compared to an AUC of 0.52 for the sum of the chlorpromazine equivalents, 0.53 for the sum of affinity constants or the sum of D2R occupancies of the individual antipsychotics (AUC=0.52).

**Discussion:**

QSP is a powerful approach to predict PD-PD interactions in the absence of any calibration set or with limited and unique data and is superior to chlorpromazine equivalents for predicting EPS liability. A major application is the simulation of pharmacodynamic interactions of comedications in clinical trials with novel compounds leading to possible better balance between the different treatment arms

